# A cross‐sectional study of Q fever in Camels: Risk factors for infection, the role of small ruminants and public health implications for desert‐dwelling pastoral communities

**DOI:** 10.1111/zph.13019

**Published:** 2023-01-05

**Authors:** Peter Holloway, Matthew Gibson, Stephen Nash, Tanja Holloway, Jacqueline Cardwell, Bilal Al Omari, Ehab Abu‐Basha, Punam Mangtani, Javier Guitian

**Affiliations:** ^1^ The Royal Veterinary College Hatfield UK; ^2^ London School of Hygiene and Tropical Medicine London UK; ^3^ Jordan University of Science and Technology Ar‐Ramtha Jordan

**Keywords:** *C. burnetii*, camels, epidemiology, Jordan, Q fever, risk factors

## Abstract

Q fever represents an important ‘neglected zoonosis’, with high prevalences recorded across the Middle East region. Among rural desert‐dwelling communities in the region, camel milk is largely consumed raw, due to perceptions of dromedaries as a uniquely clean livestock species mentioned in the Qur'an and Islamic hadith, while milk from other livestock species is usually boiled. As a result, camels present a unique public health threat among such communities from milk‐borne pathogens, including *Coxiella burnetii.* In view of this, a cross‐sectional study was conducted among dromedary herds in southern Jordan between September 2017 and October 2018, including 404 camels from 121 randomly selected herds. In addition, 510 household members associated with these herds were interviewed regarding potential high‐risk practices for zoonotic transmission. Weight adjusted camel population seroprevalence for *C. burnetii* was 49.6% (95% CI: 44.7–54.5), with evidence of maternally derived immunity in calves ≤6 months old. Adjusted herd‐level prevalence was 76.0% (95% CI 72.7–80.2). It was estimated 30.4% (144/477) of individuals consumed raw milk from infected herds monthly or more. Following multivariable logistic regression analysis, seropositive status in camels was found to be associated with increasing age, high herd tick burdens, keeping the herd together throughout the year including when calving, and owning larger (>50) sheep and goat flocks, with goats presenting a higher risk than sheep. Racing camel status was found to be protective. Socioculturally appropriate interventions aimed at raising awareness of potential risks associated with drinking raw camel milk, alongside appropriate livestock management interventions, should be considered.

**Impacts**

- *C. burnetii*, the causative agent of Q fever, is endemic at high levels among camels in Southern Jordan, which combined with widespread consumption of raw camel milk, suggest Q fever present an important public health risk among desert‐dwelling pastoral communities.
- Controlling Q fever in small ruminants (sheep and goats) is likely to contribute to reducing *C. burnetii* prevalence in associated camel populations.
- Potential control strategies include husbandry practices such as livestock tick control and separation of birthing areas as well as socioculturally appropriate interventions to raise awareness of risks from drinking raw camel milk. Use of ruminant vaccines and trials to assess them in camels should be considered.

## 1 INTRODUCTION

Q fever represents an important ‘neglected zoonosis’, which despite the presence of licensed vaccines, remains largely unrecognized and uncontrolled, particularly among lower and middle‐income countries (LMIC) where seroprevalences are often high (Vanderburg et al., 2014). The causative agent, *C. burnetii*, is an obligate gram‐negative intracellular bacterium of high tenacity, favouring hot dry conditions, with high infectivity (Maurin & Raoult, 1999). Human infections range from being asymptomatic to causing an acute non‐specific febrile illness, often with hepatitis and atypical pneumonia (van der Hoek et al., 2011). While most clinical infections are self‐limiting, some individuals go on to develop chronic disease, which may include endocarditis and fatigue (Ayres et al., 1998; Brouqui et al., 1993). These non‐specific and diverse signs and symptoms, compounded by a lack of awareness among many healthcare workers and lack of routine laboratory testing in many LMIC settings, mean that individuals presenting with clinical *C. burnetii* infection are frequently misdiagnosed (Buijs et al., 2021; Honarmand, 2012).

In ruminants, Q fever is an important production disease causing reproductive losses through abortions, stillbirths and infertility, alongside milk drop and chronic mastitis (Plummer, 2022). Bacteria are shed in high numbers through infected birth products, as well as in milk, faeces and urine (Canevari et al., 2018). Livestock and human infections occur largely via inhalation of contaminated dust particles, including infected tick faeces, as well as through contact with infected birthing products and from infected tick bites (Angelakis & Raoult, 2010). However, zoonotic transmission via consumption of infected raw dairy products is also known to occur (Signs et al., 2012). While the zoonotic impact of *C. burnetii* infection in small ruminant and cattle populations has been widely reported, the potential role of camels in zoonotic transmission of Q fever has until recently, remained largely unexamined, particularly in the Middle East region, where favourable conditions for the pathogen exist (Browne et al., 2017; Devaux et al., 2020; Hussien et al., 2017; Larson et al., 2019). The widespread consumption of raw camel milk across the Arab world, due to the perceptions of camels as uniquely clean livestock with mention in the Qur'an and Islamic hadith, means that camels present a unique public health threat (Al‐Ghāshiyah; Qilaba; Galali & Al‐Dmoor, 2019). A population‐level seroprevalence of 24% was recently reported in Jordan, with seroprevalences of 52% and 35% reported among hospitalised patients with fever of unknown origin, in neighbouring Saudi Arabia and Egypt respectively, suggesting Q fever presents an important public health threat within the region.(Abbass et al., 2020; Almogren et al., 2013; Obaidat et al., 2019)

To improve understanding of the epidemiology and potential zoonotic risks posed by Q fever in camels, we conducted a large‐scale epidemiological survey among camel herds in southern Jordan, largely owned by desert‐dwelling Bedouin communities. This population is considered likely to be representative of analogous Bedouin and pastoral communities in the wider region, where larger flock or herd sizes are indicative of higher socioeconomic status. The objectives of the study were to: (i) estimate the prevalence of *C. burnetii* in the camel population in southern Jordan (ii) identify potential transmission pathways for *C. burnetii* infection in camels, particularly regarding the role of small ruminants, and (iii) assess the potential public health risk associated with these herds through consumption of raw milk and other activities.

## 2 METHODS

### 2.1 Study design and study population

**FIGURE 1:**
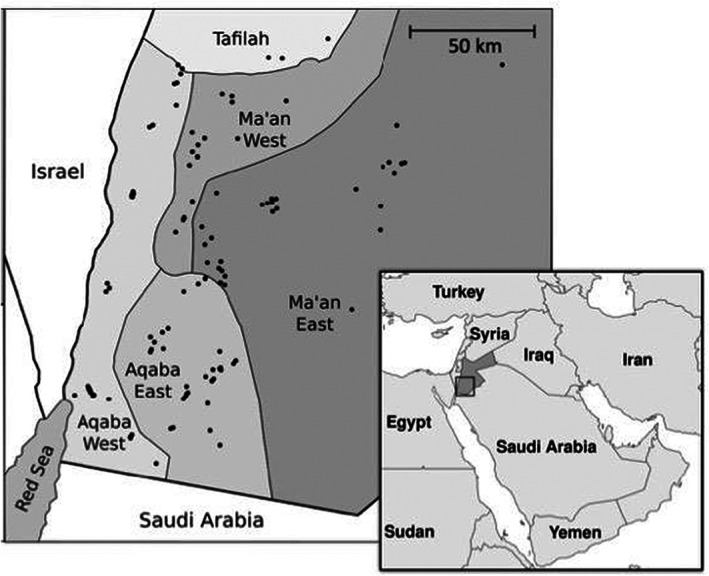
Location of 121 camel herds sampled in southern Jordan October 2017 to October 2018 (due to local grazing movements there were three herds selected from the MoA list for Ma'an west that were sampled in the neighboring region, Tafilah. Results from these herds were attributed to Ma'an west)

A cross‐sectional study was conducted between 28th October 2017 and 11th October to 2018, in Aqaba and Ma'an governorates of southern Jordan, an area of approximately 40,000 km^2^ and 8000 camels (based on MoA data) (Figure 1). Probabilistic sampling was conducted using camel owner lists supplied by the Ministry of Agriculture (MoA) according to four local administrative areas (Aqaba east, Aqaba west, Ma'an east and Ma'an west). The MoA records livestock numbers owned per registered individual in each administrative region, including camels, with these records updated annually. While it is possible that some individuals who own camels may have been omitted from this list, all individuals contacted from these lists owned camels, or had done so at the time of the list had been compiled, with numbers owned largely reflecting MoA records.

Based on an expected median herd size of 12, an expected prevalence of 35% and a confidence level of 95%, and in order to facilitate owner compliance, no more than 12 camels were sampled per herd, and in herds of <12 all camels were sampled, subject to accessibility and owner permissions. The formula used to detect at least one positive animal was: *K* = [1−(1−p1/*d*)][*N*−½(*d*−1)]. Two standardised structured questionnaires regarding potential risk factors for *C. burnetii* infection, in camels and humans respectively, were administered in the local dialect on Android tablets, using the application Open Data Kit (ODK), among herd owners and their household members. All camels included in the study were clinically examined by a veterinary surgeon to assess general health and the presence of ticks (yes/no), prior to collection of a blood sample, from which serum was extracted.

### 2.2 Laboratory methods

Blood samples were collected in 8 ml serum vacutainer tubes, transported in cool boxes and centrifuged at 2000 RPM for 10 min, followed by serum collection and storage at −20°C. Laboratory testing was performed at the Diagnostic Laboratory, Veterinary Health Centre, Jordan University of Science and Technology, Ar‐Ramtha, Irbid, using an indirect ELISA (ID Screen Q fever Indirect Multi‐species, IDVet), according to the manufacturer's recommendations (ID Screen® Q Fever Indirect Multi‐species).

### 2.3 Statistical analysis

We calculated seroprevalence estimates, weighted according to sample size, relative to the estimated camel population, based on MoA data for each sub‐region. Regression models were built for identification of risk factors, with camels ≤6 months of age excluded from analyses due to the potential influence of maternally derived antibodies.

Univariable analyses were conducted, using mixed‐effects logistic regression to adjust for herd‐level random effects, with camel serological status considered a binary outcome. All potential risk factors were analysed as categorical variables, with the exception of camel age, altitude of the holding, and small ruminant flock size which were analysed as continuous variables. Season was not considered for analysis due to the non‐longitudinal nature of the study and likely correlation with sample location.

Variables associated with the outcome with a *p*‐value <0.2 were considered for inclusion in the multivariable models, with the exception of any variables missing more than 10% of their values. Collinearities between variables were examined using the Pearson *R* coefficient and a threshold of 0.4, with collinear variables excluded from the same multivariable model. Multivariable models were constructed using a backwards stepwise method, with the least significant variable removed at each step while *p* > 0.1, unless the variable was considered an a priori factor (sex and age) or the removal of the variable demonstrated a significant effect on the other variables (a change in log odds >20%), with model building then repeated using a forwards stepwise method.

The herd‐level prevalence of *C. burnetii* (the proportion of herds containing at least one camel with *C. burnetii* antibodies) was estimated, taking into account the uncertainty arising from sampling only a proportion of each herd (the proportion being different in each herd). Based on the method described by Beauvais et al. (Beauvais et al., 2016), a frequency distribution of within‐herd prevalence was calculated for each herd and then multiplied by the number of camels in the herd. This was used in a Bayesian computation with herd sample results, giving a discrete probability distribution for positives within a herd. Each herd was then simulated as being positive or negative using a random sample from a binomial distribution. This was repeated 10,000 times to create an uncertainty distribution, where the 2.5th and 97.5th percentiles gave a 95% credible interval and the 50th percentile gave the most likely herd‐level prevalence.

The number of individuals living in households with Q fever positive herds was compared against questionnaire data relating to potential pathways for *C. burnetii* zoonotic transmission, by calling a herd positive when there was at least a 50% probability (with 95% confidence) of the herd having at least one positive animal, using Bayesian probability. This figure was used to a calculate the percentage of the sample population likely to have been exposed to potential *C. burnetii* transmission via high‐risk practices.

All statistical analyses were performed in *R* (version 3.5.6) with mixed‐effects models generated using the glmer function of the package lme4 (version 1.1–23).

### 2.4 Informed consent

Informed consent was obtained from all participating camel owners and household members at the time of sampling. Institutional and national guidelines for care, use, and handling of animals were followed at all times, with institutional ethical review board approval by the Royal Veterinary College (ref. no. 2016/1551), London School of Hygiene and Tropical Medicine (ref. no. 14472) and Jordan University of Science and Technology (ref. no. 9/107/2017).

## 3 RESULTS

Blood samples were collected from 404 camels in 121 herds, with an average of 3.3 camels sampled per herd (median herd size 9, IQR 4–17). The questionnaire regarding potential risk factors for infection in camels was administered to all 121 herd owners, while the questionnaire regarding potential high‐risk practices for human infection was administered to 510 members of camel‐owning households (which included the 121 herd owners). Camel numbers sampled were: Ma'an east 90 (29 herds), Ma'an west 69 (21 herds), Aqaba east 147 (36 herds) and Aqaba west 90 (35 herds). MoA records described an estimated 1909 camels (138 herds) in Ma'an East, 1405 camels (127 herds) in Ma'an West, 3563 camels (198 herds) Aqaba East and 873 camels (119 herds) in Aqaba West. Model outcomes were thus weighted for each region by 2.02, 0.65, 0.66, 0.80, respectively.

**FIGURE 2:**
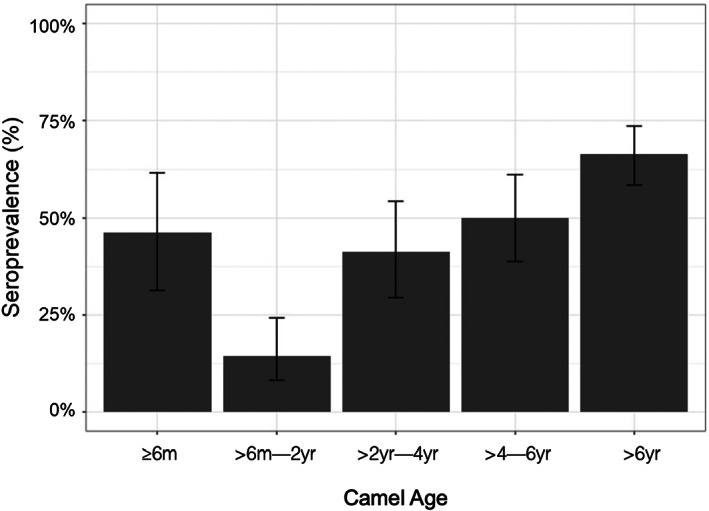
*Coxiella burnetii* seroprevalence among camel populations in southern Jordan, October 2017 to October 2018, stratified by age

Of the 404 camels sampled there were 8 samples with insufficient serum. Of the remaining 396 samples, 189 were seropositive for *C. burnetii*, giving an unadjusted seroprevalence of 47.7% and a weighted seroprevalence of 49.6% (95% CI: 44.7–54.5). Of these, 39 camels were aged ≤6 months with 18 seropositive (46.2%), OR 5.1; 95% CI 2.1–12.8; *p* < 0.01 compared with camels >6 months–2 years of age of whom 14.5% (11 positive/76 total) were seropositive, with seroprevalence then increasing with age (Figure 2). Following removal of calves ≤6 months old from the data set, the adjusted seroprevalence was 49.3% (95% CI: 44.0–54.6) among 119 herds. Ticks were observed on 226 (55.9%) of camels sampled.

Descriptive statistics and univariable model results are presented in Table 1 and Figure 2. Significant correlations, where *R* > 0.4, were identified between the variables ‘region’ & ‘altitude’, between ‘herd owner has small ruminants (linear per 10)’ and ‘herd owner has >50 sheep’ and ‘herd owner has >50 goats’, and between ‘closed herd’ and ‘contact with local camels’ (inverse).

**TABLE 1:** Characteristics of study sample and univariate associations between potential risk factors and *Coxiella burnetii* seropositivity in camel populations in southern Jordan, October 2017 to October 2018 (due to the potential influence of maternal immunity camels ≤6 m have been excluded from all variables except age)

Variable	Category	Total (missing)	Positive	(%)	OR (95% CI)	*p* value
Region	Aqaba	226	80	35%	6.73 (2.20–20.58)	<0.01
	Ma'an	131	91	69%		
Sub‐region	Aqaba East	140	40	29%	1.00	<0.01
	Aqaba West	86	40	47%	4.08 (1.09–15.29)	
	Ma'an East	67	36	54%	5.78 (1.53–21.78)	
	Ma'an West	64	55	86%	68.44 (9.87–474.67)	
Altitude	(per 1000 m)	–	–	–	2.62 (0.75–9.21)	0.13
Sex	Male	80	23	29%	1.00	0.04
	Female	277	148	53%	2.49 (1.04–5.97)	
Age	0–6 months	39	18	46%	–	–
	6 months–2 years	76	11	14%	1.00	<0.01
	2 years–4 years	58	24	41%	6.00 (1.61–22.37)	
	4 years–6 years	74	37	50%	12.79 (3.47–47.10)	
	>6 years	149	99	66%	24.85 (7.03–87.82)	
Age	(per year)	–	–	–	1.28 (1.15–1.42)	<0.01
Herd size	1–10	158	71	45%	1.00	0.43
	10–20	74	40	54%	2.29 (0.56–9.37)	
	>20	125	60	48%	1.84 (0.49–6.92)	
Herd size	(per 10 camels)	–	–	–	0.99 (0.80–1.23)	0.96
Number of camel herds nearby (within 15 min drive)	≤20	254	127	50%	0.75 (0.21–2.61)	0.65
	>20	103	44	43%		
Herd is kept together as single group throughout the year	No	106	35	33%	6.93 (2.25–21.41)	<0.01
	Yes	196	115	59%		
Contact with local herds	No	193	90	47%	2.17 (0.72–6.57)	0.17
	Yes	164	81	49%		
Contact with distant herds (transhumance)	No	169	79	47%	2.02 (0.68–6.01)	0.21
	Yes	188	92	49%		
New camels are purchased	No	233	110	47%	0.84 (0.27–2.58)	0.76
	Yes	124	61	49%		
Some form of quarantine is practiced following purchase	No	100	50	50%	0.59 (0.04–7.94)	0.69
	Yes	24	11	46%		
Camels are borrowed for breeding purposes	No	204	96	47%	1.80 (0.60–5.37)	0.30
	Yes	153	75	49%		
Camels are lent for breeding purposes	No	199	97	49%	1.87 (0.61–5.69)	0.27
	Yes	158	74	47%		
Camel is a racing camel	No	271	146	54%	0.15 (0.03–0.63)	0.01
	Yes	33	3	9%		
Closed herd^a^	No	273	139	51%	0.29 (0.08–1.08)	0.07
	Yes	84	32	38%		
Herd tick burden high (all camels sampled had ticks)	No	216	72	33%	10.25 (3.31–31.69)	<0.01
	Yes	141	99	70%		
Tick treatment: acaracide washes/year	0	253 (15)	132	52%	1.00	0.15
	1–3	68	24	35%	0.51 (0.14–1.86)	
	>3	21	2	10%	0.07 (0.00–1.38)	
Tick treatment: >3 acaracide washes/year	No	321 (15)	156	49%	0.08 (0.00–1.62)	0.10
	Yes	19	2	10%		
Tick treatment: Ivermectin injections/year	0	227 (15)	101	44%	1.00	0.61
	0–3	98	51	52%	1.67 (0.49–5.65)	
	>3	17	6	35%	0.56 (0.05–6.90)	
Tick treatment: >3 Ivermectin injections/year	No	325 (15)	152	47%	0.48 (0.04–5.84)	0.57
	Yes	17	6	35%		
Herd owner owns small ruminants^b^	(per 10 animals)	–	–	–	1.05 (1.01–1.10)	0.02
Herd owner owns sheep	No	105	50	48%	0.93 (0.28–3.07)	0.91
	Yes	252	121	48%		
Herd owner owns goats	No	46	23	50%	0.51 (0.11–2.38)	0.39
	Yes	311	148	48%		
Herd owner owns >50 sheep	No	260	107	41%	5.48 (1.61–18.66)	0.01
	Yes	97	64	66%		
Herd owner owns >50 goats	No	259	99	38%	8.64 (2.51–29.73)	<0.01
	Yes	98	72	73%		
Dog(s) present	No	42	19	45%	0.74 (0.15–3.77)	0.72
	Yes	315	152	48%		
Cat(s) observed	No	55	27	49%	1.94 (0.48–7.72)	0.35
	Yes	302	144	48%		
Rats / mice observed daily or weekly	No	217	97	45%	2.27 (0.76–6.82)	0.14
	Yes	140	74	53%		
Drinking source: Spring water	No	308	151	49%	0.95 (0.19–4.66)	0.95
	Yes	49	20	41%		
Drinking source: Irrigation reservoir	No	327	157	48%	4.93 (0.57–42.76)	0.15
	Yes	30	14	47%		
Drinking source: Tanker	No	241	115	48%	1.47 (0.45–4.74)	0.52
	Yes	116	56	48%		
Drinking source: Tap	No	192	96	50%	0.87 (0.29–2.63)	0.80
	Yes	165	75	45%		
Drinking source: Well	No	271	123	45%	2.15 (0.60–7.77)	0.24
	Yes	86	48	56%		
Water source not shared with the household	No	259	123	47%	0.63 (0.11–3.47)	0.58
	Yes	98	48	49%	0.61	
Shared water source^c^	Open *ad lib*	53	24	45%	1.00	0.80
	Not shared	98	48	49%	0.61 (0.11–3.47)	
	Trough only	206	99	48%	0.59 (0.12–2.89)	

^a^ Closed herd = herds answering no to all of the following variables; borrowing, lending, purchasing, racing, contact with local and/or distant herds.

^b^ Up to flock size ≤500 (5 flocks of recorded flock size >1000 were excluded due to reasonable suspicion of inaccuracy).

^c^ Open ad lib = irrigation reservoir and/or spring water sources used, Household only = water source not shared between household and herd, Trough only = tanker and/or tap and/or well sources used only.

Multivariable model results are shown in Table 2, with the variable, ‘camel is a racing camel’ found to be protective (OR_adj_ 0.14; 95% CI 0.03–0.62; *p* = 0.012). There was evidence of positive association between *C. burnetii* seropositivity and increasing age, per year (OR_adj_ 1.26; 95% CI 1.14–1.42; *p* < 0.001), high herd tick burden (all camels sampled from the herd had ticks) (OR_adj_ 7.90; 95% CI 2.50–19.29; *p* < 0.001), herd kept as a single group throughout the year (OR_adj_ 5.93; 95% CI 1.52–10.77; *p* = 0.006). While simply owning sheep and goats was not significantly (<0.05) associated with risk, increasing flock size (linear, per 10) (OR_adj_ 1.10; 95% CI 1.00–1.09; *p* = 0.037), and owning larger flocks were associated: flock size >50 goats (OR_adj_ 8.78; 95% CI 2.04–18.06; *p* = 0.001) and flock size >50 sheep (OR_adj_ 5.22; 95% CI 1.20–11.24; *p* = 0.024). Due to significant collinearity (*R* > 0.4), these latter two variables were analysed in separate models, in place of the variable ‘herd owner has small ruminants’ (linear, per 10); all other variables maintained in the final model continued to demonstrate significant association (*p* < 0.05) with *C. burnetii* seropositivity. Also due to collinearity (*R* > 0.4), the variables ‘closed herd’ and ‘contact with local herds’ were included in separate multivariable models, though neither was maintained in the final model.

**TABLE 2:** Multivariable associations between potential risk factors and *Coxiella burnetii* seropositivity in camel populations in southern Jordan, October 2017 to October 2018 (due to the potential influence of maternal immunity camels ≤6 m have been excluded)

Variable	Category	A‐priori adjusted OR (95% CI)^a^	*p* value	Fully adjusted OR (95% CI)^b^	*p* value
Age	(per year)	1.26 (1.14–1.40)	<0.001	1.26 (1.14–1.42)	<0.001
Herd tick burden high (all camels sampled had ticks)	Yes	7.90 (2.69–23.18)	<0.001	7.90 (2.50–19.29)	<0.001
Herd kept as single group all year	Yes	5.93 (2.06–17.10)	<0.001	5.93 (1.52–10.77)	0.006
Herd owner also owns small ruminants^e^	(per 10)	1.05 (1.01–1.09)	0.017	1.04 (1.00–1.09)	0.037
Camel is a racing camel	Yes	0.14 (0.03–0.63)	0.010	0.14 (0.03–0.62)	0.012
Sex	Female	1.62 (0.68–3.86)	0.281	1.62 (0.36**–**2.35)	0.874
Altitude^c^	(per 1000 m)	1.90 (0.58–6.21)	0.286	–	–
Contact with local herds	Yes	1.86 (0.67–5.20)	0.237	–	–
Closed herd^d^	Yes	0.27 (0.08–0.93)	0.039	–	–
Tick treatment: >3 acaracide washes/year.	Yes	0.16 (0.01–2.88)	0.215	–	–
Rats/mice observed daily or weekly	Yes	2.19 (0.79–6.07)	0.131	–	–
Drinking source: Irrigation reservoir	Yes	7.23 (0.98–53.45)	0.053	–	–
Herd owner owns > 50 goats^e^	Yes	8.78 (2.78–27.77)	0.010	8.78 (2.04–18.06)	0.001
Herd owner owns > 50 sheep^e^	Yes	5.22 (1.66–16.38)	0.005	5.22 (1.20–11.24)	0.024

^a^ Adjusted for a‐priori variables; age and sex.

^b^ Adjusted for; Sex, Age, Herd ticks burden high, Herd kept as a single group all year, Herd own owns small ruminants, Camel is a racing camel.

^c^ Due to significant collinearity (*R* > 0.4) between ‘region’ and ‘altitude’, altitude was chosen over region for inclusion in the final model.

^d^ Closed herd = herds answering no to; ‘borrowing, lending, purchasing, racing, contact with local and/or distant herds.’ Due to significant collinearity (*R* > 0.4), the variables ‘closed herd’ and ‘contact with local herds’ were included in separate multivariable models, though neither variable was maintained in the model.

^e^ Due to significant collinearity (*R* > 0.4) between the variables ‘Herd owners owns small ruminants (linear, per 10)’, ‘Herd owner owns >50 sheep’ and ‘Herd owner owns >50 goats’, these variables were included in separate multivariable models. In these models, all variables listed continued to demonstrate significant association (*p* < 0.05) with *C. burnetii* seropositivity.

At the herd level, 76.0% (95% credible interval 72.7–80.2) were estimated as being positive for Q fever (having at least one *C. burnetii* seropositive camel present in the herd). It was estimated that 30.4% (145/477) of individuals in camel‐owning households were frequently (monthly or more often) drinking raw milk from (their own) Q fever positive herds. In addition, in the past year, 18.8% (96/510) of individuals had been involved in calving camels, 16.7% (85/510) in handling birthing products, 16.5% (84/510) in cleaning camel pens, 7.6% (39/510) in slaughtering camels, 2.6% (13/494) in handling raw camel meat and 10.4% (53/510) had been bitten by ticks (from their own *C. burnetii* positive herds) (Table 3).

**TABLE 3:** Percentage of study population exposed to potential *Coxiella burnetii* transmission pathways, using a Bayesian method to predict positive herds with 95% confidence, among camel owning households in southern Jordan, October 2017 to October 2018 (due to the potential influence of maternal immunity, exposure to seropositive camels ≤6 m was not included)

Variable	Category	No. of individuals within *C. burnetii* +ve herd households (missing)		No. of individuals within *C. burnetii* −ve herd households (missing)		No. of individuals exposed/total study population	% of camel owning population exposed
Drink raw camels' milk from own herd	Yes	145	(26)	47	(7)	145/477	30.4%
	No	207		78			
Clean own camel pens	Yes	84		39		84/510	16.5%
	No	294		93			
Calve own camels	Yes	96		31		96/510	18.8%
	No	282		101			
Handle camel afterbirth	Yes	85		29		85/510	16.7%
	No	293		103			
Slaughter own camels	Yes	39		14		39/510	7.6%
	No	339		118			
Handling raw meat from own camels	Yes	13	(14)	7	(2)	13/494	2.6%
	No	351		123			
Bitten by ticks	Yes	53		6		53/510	10.4%
	No	325		126			

## 4 DISCUSSION

High seroprevalences of *C. burnetii* are reported in human and livestock populations in the Middle East, where a hot dry climate and open deserts, combined with high, localised small ruminant populations, provide favourable conditions for transmission via dust and wind (Ergas et al., 2006; Lafi et al., 2020). However, while the zoonotic risk of Q fever from small ruminants and cattle is well established, the potential risk from camels in the region is poorly understood. Deeply held religious and cultural beliefs regarding the healing benefits of camels' milk and urine – meaning that among rural communities in the Arab world, camel milk is usually consumed raw, while the milk from sheep and goats is usually boiled (Abdel Gader & Alhaider, 2016; Galali & Al‐Dmoor, 2019). However, high *C. burnetii* seroprevalences among camel populations in the region (Selim & Ali, 2020), and detection of *C. burnetii* in raw camel milk samples collected in the region (Esmaeili et al., 2019), suggest consumption of raw camel milk may present an important determinant for Q fever among human populations in the Middle East, alongside other potential transmission routes.

In desert‐dwelling pastoral communities, camels and small ruminants are commonly kept together, with potential for pathogen transmission between species (Selmi et al., 2018). This means that zoonotic pathogens such as *C. burnetii*, which can be transmitted from small ruminants to camels, present a risk to households owning mixed flocks/herds of small ruminants and camels, via the consumption of raw camel milk, even when milk from other livestock species is boiled. Lafi et al. report *C. burnetii* seroprevalences of 27.0% and 43.3% among sheep and goats in Jordan, respectively (Lafi et al., 2020). While Q fever seroprevalences have been reported in camel populations in Tunisia (44.4%), Egypt (21.9%) and Iran (28.7%), our study is the first to report a seroprevalence estimate for camels in Jordan (49.6%; 95% CI: 44.7–54.5) (Janati Pirouz et al., 2015; Selim & Ali, 2020; Selmi et al., 2018).

In the study population, almost a third of camel owners and their households were found to be frequently drinking raw camel milk from *C. burnetii* positive herds. This indicates a clear public health risk from Q fever in camels in the region – alongside other risk‐associated camel‐engagement activities such as cleaning pens, handling afterbirth, facilitating calving, slaughtering, handling raw meat or camel‐tick bites (Devaux et al., 2020; Mohammadpour et al., 2020). In addition, high *C. burnetii* seroprevalences in camels suggest likely production losses through infertility, abortions, still‐births, weak off‐spring, milk drop or chronic mastitis, all with important economic impact (Plummer, 2022).

Where camels and small ruminants were owned together, simply owning sheep and/or goats was not found to be associated with a significant increase in *C. burnetii* seroprevalence in these camel herds. However, larger small ruminant flock sizes were significantly associated with higher *C. burnetii* seroprevalence in camel herds associated with these flocks. Small ruminant flock sizes of >50 sheep or goats were associated with increased *C. burnetii* seroprevalence in associated herds. In addition, the risk associated with large goat flocks was greater than that associated with large sheep flocks. This is consistent with previous findings identifying goats as posing a greater risk for *C. burnetii* transmission than sheep (Lafi et al., 2020; Vellema et al., 2021).

Our study findings suggest that controlling Q fever in small ruminant populations is likely to be important in reducing *C. burnetii* seroprevalence in associated camel populations. An effective commercially available livestock vaccine has been used extensively in the Netherlands and its potential use among small ruminants and cattle in Jordan, and the wider region, should be considered (European Medicines Agency; Hogerwerf et al., 2011). This product is currently not licensed for use in camels; however efficacy studies regarding potential use in camels should be considered. This study is the first to demonstrate evidence of maternal antibodies to *C. burnetii* in camels, lasting until approximately 6 months of age. This suggests that vaccination should potentially be delayed until after 6 months of age, with further research required*. C. burnetii* seroprevalence increased significantly with camel age, consistent with increased risk of exposure over time and antibodies being long‐lasting, in keeping with other studies (Selim & Ali, 2020).

Due to the high shedding known to occur in small ruminant birthing and aborted materials, small ruminant flocks should be separated from camel herds during the lambing/kidding period where possible (Devaux et al., 2020). Separate birthing areas for small ruminants and camels are advisable, due to *C. burnetii's* ability to form tenacious spore‐like cell variants, capable of remaining in the environment for more than a year, (Devaux et al., 2020; Plummer, 2022). In addition, because the pathogen can travel long distances via the wind, these areas should be as far apart as possible, and moved regularly. The practice of keeping herds together as a single group throughout the year was associated with significantly higher *C. burnetii* seroprevalences in studied herds. This is likely explained by the increased exposure to infected camel birthing products in these herds, compared to herds where pregnant females are removed prior to calving (Devaux et al., 2020). This suggests removal of pregnant females from the herd prior to calving to be a potentially important management intervention in reducing *C. burnetii* transmission between camels. The importance of herd owner hygiene through hand washing (and use of disinfectant foot baths where practical) after working with small ruminants at parturition time, should also be stressed (Bardenstein et al., 2021; Musallam et al., 2016; Signs et al., 2012).

Ticks are known to play an important role in *C. burnetii* transmission in livestock populations globally (Devaux et al., 2020). In our own study, a high herd tick burden (meaning all camels sampled from the herd had ticks) was significantly associated with higher herd *C. burnetii* seroprevalences. *C. burnetii* has been identified in camel ticks across Africa and the Middle East, particularly in *Hyalomma ssp, Amblyomma spp*. and *Rhipicephalus spp*. (Getange et al., 2021; Mumcuoglu et al., 2022). This suggests that aggressive tick control, using frequent acaracide washes (for example monthly or every 2 months), plays an important role in Q fever control among camel populations in the region, with further research required (el‐Azazy, 1996). In addition, parallel tick control in small ruminant populations associated with these herds, for example through quarterly dipping, could also be expected to offer a protective effect.

Racing animals are widely owned among camel‐owning communities in southern Jordan, with seroprevalence significantly lower among these animals. This is likely explained by separation of racing camels from the main herd, and small ruminant flocks, for training and management purposes, rather than socioeconomic factors. However, parturition during racing lifetime is limited, and racing camels are not usually used in milk production, meaning lower *C. burnetii* seroprevalences are of limited public health impact.

This study is subject to limitations. First, household members, were not tested for the presence of *C. burnetii* antibodies. It is recommended that future studies be conducted among camel owning communities in the region to determine seroprevalence and risk factors for infection, alongside potential screening of febrile patients in local healthcare settings. Similarly, small ruminant flocks associated with these herds should also be tested. Second, tick burdens per camel were not enumerated or speciated, meaning animal level tick burdens could not be related to Q fever serostatus at an individual level. Third, the ELISA test used has only been validated for use in sheep, goats and cattle, meaning lack of sensitivity and specificity estimates for use in camels precluded adjustment of seroprevalence for test performance.

In conclusion, the high seroprevalence of *C. burnetii* in camel herds in southern Jordan coupled with frequent consumption of camel milk and animal husbandry practices where exposure to contaminated environment is high, indicates a clear public health risk. To reduce the zoonotic risk, and to reduce potential production losses, the following targeted management interventions aimed at limiting the transmission of *C. burnetii* in camels should be considered: (i) removal of breeding camels from the herd prior to calving, (ii) creating separate birthing areas for small ruminants and camels, as far apart as possible and moved regularly, (iii) promoting owner biosecurity measures that include hand hygiene (and use of disinfectant foot baths where practical) after working with recently calved or aborted livestock or after handling aborted material. Control measures in small ruminants are of particular importance when managing camel herds alongside larger flocks, particularly regarding goats. Potential licensing and use of existing *C. burnetii* vaccines in Jordan, for use in small ruminants (and cattle), should be considered. In addition, efficacy studies regarding the use of such vaccines in camels should be conducted.

Given the high percentage (over 30%) of individuals in camel‐owning households drinking raw camel milk from *C. burnetii* positive herds, educational efforts to promote boiling of camels' milk should be encouraged. However, in view of the profound cultural barriers likely to be encountered, detailed ethnographic studies to identify public health interventions that are culturally appropriate should first be conducted. In summary, Q fever represents an important zoonosis in the Middle East region and beyond, with high population seroprevalences previously recorded. High *C. burnetii* seroprevalences identified in camels, alongside widespread engagement in high‐risk camel‐associated practices, including consumption of raw milk, suggest camels likely present a high‐risk species for human infection, with culturally appropriate veterinary and public health interventions urgently needed.

## CONFLICT OF INTEREST

The authors declare that they have no conflict of interest.

## Data Availability

Data available on request from the authors. The data that support the findings of this study are available from the corresponding author upon reasonable request.
